# Association between screen time and depressive and anxiety symptoms among Chinese adolescents

**DOI:** 10.3389/fpsyt.2025.1428885

**Published:** 2025-05-06

**Authors:** Jue Xu, Hanmin Duan, Kang Qin, Bing Liu

**Affiliations:** ^1^ Hangzhou Center for Disease Control and Prevention (Hangzhou Health Supervision Institution), Hangzhou, Zhejiang, China; ^2^ Department of Big Data in Health Science, School of Public Health, Center of Clinical Big Data and Analytics, The Second Affiliated Hospital, Zhejiang University School of Medicine, Hangzhou, China

**Keywords:** adolescents, screen time, mental disorders, sleep duration, mediator analysis

## Abstract

**Background:**

Although many studies have explored the relationship between screen time and depression and anxiety symptoms, there is still a lack of in-depth research in Chinese adolescents who are stressed and sleep-deprived. This research aims to investigate this link and examine the role of sleep duration as a mediating factor.

**Method:**

Data were collected from 3,307 students from four districts and counties of Hangzhou, Zhejiang Province, China, using a multi-stage random cluster sampling method and self-administered questionnaires. The study applied linear regression to investigate the relationship between screen time and depression and anxiety symptoms, and mediation analysis to understand how sleep duration might influence this relationship.

**Results:**

In total, approximately 25.5% of the teenagers had more than 2 hours of screen time (33.56% for electronic devices and 17.46% for television), and the average score was 5.942 ± 0.085 for depression symptoms and 4.521 ± 0.076 for anxiety symptoms. The linear regression analysis showed that longer electronic device use (*β*=0.601, 95%CI: 0.265 to 0.937) and television usage (*β*=0.751, 95%CI: 0.346 to 1.156) were positively associated with depression symptoms, and electronic device use was also positively associated with anxiety symptoms (*β*=0.471, 95%CI:0.159 to 0.784). The study found that sleep duration plays a partial mediating role between screen time and mental disorders. For electronic device use, the effect was -27.50% in depression and -44.01% in anxiety symptoms; for television use, the effect was -42.70% in depression symptoms.

**Limitations:**

The cross-sectional study design could not prove causation.

**Conclusions:**

The association between screen time and depression and anxiety symptoms in adolescents was positive, and sleep duration acted as a mediator.

## 1 Introduction

The World Health Organization (WHO) reported that children aged 10–19 years constituted 17% of the world’s population (1), and adolescence is a unique time of development when physical, emotional, and social changes are taking place. Furthermore, exposure to poverty, abuse, or violence can make adolescents vulnerable to mental disorders (2, 3). In today’s fast-paced and challenging social environment, adolescent mental health issues have become a public health concern worldwide, with anxiety and depression symptoms particularly prominent (4). The WHO defines depression as a depressive disorder or depression, and it is a common mental health condition that can happen to anyone. It is characterized by a low mood or loss of pleasure or interest in activities for long periods of time (5). In addition, the WHO has the following description of anxiety: anxiety disorders are the world’s most common mental disorders, affecting 301 million people in 2019. Everyone experiences anxiety to some extent, but people with anxiety disorders often feel intense fear and worry. This feeling is usually accompanied by physical tension and other behavioral and cognitive symptoms. Anxiety is difficult to control and can cause great distress. If not treated promptly, it will last for a long time. Anxiety disorders can interfere with daily activities and may damage a person’s family, social, school, or work life (6). It is reported that the estimated regional and global prevalence rates of any depressive disorders and major depressive disorders in children and adolescents were 2.6% and 1.3% respectively (7). A nationally representative survey of adolescents (aged 13–18) found that anxiety (31.9%) was the most common mental disorder in terms of lifetime prevalence in the United States (8) and the prevalence of anxiety disorders among adolescents in Canada ranges from 12% to 20% (9). A meta-analysis of a sample of Chinese adolescents showed a prevalence of 24.3% for depression and 24.0% for anxiety (10), thus, the data shows that the prevalence of anxiety disorders among adolescents in China is higher than in Canada. The prevalence of depression among adolescents in Europe varies from country to country, ranging from 7.1% to 19.4%, and the 12-month prevalence of anxiety disorders among adolescents in Europe is approximately 3%–10% (11). Studies in Australia have shown that the 12-month prevalence of depressive disorders in adolescents is approximately 6%–7%, and the 12-month prevalence of anxiety disorders is approximately 10%–15% (12). Furthermore, a study has indicated that the prevalence of depressive disorders in Brazilian adolescents is approximately 10%–12%, and the prevalence of anxiety disorders is approximately 8%–10% (13). Depression and anxiety symptoms can affect children’s and adolescents’ performance at school or work and cause dysfunction in interactions with family and peers, which can negatively impact an adolescent’s developmental trajectory (14, 15), and even increase the risk of suicidal behavior, alcohol and substance use disorders, and learning difficulties later in life (16).

Screen time refers to watching or using anything with a screen, including mobile phones, computers, and televisions (17). Rapid advances in screen technology have increased the amount of time children and teenagers spend in front of electronic screens (18). The use of smartphones and other small Internet-enabled mobile devices has had an alarming increase (19). Worldwide, 80% of adolescents are physically inactive, and many have more than 2 hours of screen time per day (20). In the United States, data from a nationally representative survey showed that from 2009 to 2015, teenagers’ average daily screen time increased by 0.7 hours (21), and in terms of the distribution of screen time, American teenagers mainly spend time on video games and social media sites (22). In China, a national survey in 2016 found that more than 36% of Chinese teenagers spend more than 2 hours a day in front of screens (23), and the screen time continues to increase (24). Some researchers have found that among adolescents, there exists a positive association between screen time and mental disorders (25, 26), and higher screen time was associated with some chronic diseases such as diabetes, overweight, and all-cause mortality (27, 28). Moreover, the cross-sectional questionnaire data of a cohort study showed that at age 14, social media use was associated with depressive symptoms, and the correlation was stronger in girls (29). Genes may explain part of the link between screen time and mental problems in children and adolescents (30). The link between screen time and mental disorders in adolescents has also been discovered in recent studies (31, 32).

Presently, bad diets, early life adversity, and social environments, such as family environment, social relations, and school environment, will have an impact on adolescent mental health (33, 34). However, some risk factors are immutable, or influenced by multiple cultural factors and individual metabolism, and it is not easy to intervene, such as for bad diets (35, 36). Thus, it is necessary to identify some modifiable risk factors and intervene to reduce the incidence of adolescent psychological problems. Given that screen time is an alterable risk factor for depression and anxiety symptoms, investigating this relationship could offer insights into mental disorder prevention in adolescents. A study demonstrated that the use of screen devices, such as TVs, mobile phones, and tablets, may affect sleep and in children aged 4–18 years, high TV use was associated with delayed bedtime and shorter self-reported sleep duration (37). There was also an association between the use of interactive devices and shorter self-reported sleep duration (38–40). Two possible mechanisms can account for these associations. First, it can be attributed to the use of the device itself, including factors such as sleep displacement, mental arousal, and emissions of blue light from the screen. Secondly, exposure to radio frequency electromagnetic fields (RF-EMF) may also play a role (41). Existing evidence suggests that sleep disorders and sleep deprivation are associated with functional deficits in various psychological, interpersonal, and physical health indicators (42, 43). For example, adolescents with sleep disorders have reported more symptoms of depression, anxiety, anger, inattention, behavioral problems, drug and alcohol use, impaired academic performance, and suicidal thoughts and behaviors (44, 45). They are also reported to experience more fatigue, have less energy, perceive a deterioration in their health status, and suffer from symptoms such as headaches, stomachaches, and backaches (46, 47). However, these studies did not explore the mediating role of sleep between screen time and depression and anxiety symptoms in Chinese adolescents. Therefore, our study also considers the potential role of sleep time and aims to explore the mediating effect of sleep duration between screen time and mental disorders, offering a foundation for disrupting the potential causal chain linking screen time and depression and anxiety symptoms.

## 2 Hypotheses

Longer screen time causes higher depression and anxiety symptom scores, and sleep duration has a mediation effect and reduces the severity of mental disorders.

## 3 Methodology

### 3.1 Participants

The Youth Risk Behavior Surveillance System (YRBSS), a project by the Zhejiang Provincial Center for Disease Control and Prevention in China, aims to understand the epidemiological characteristics of adolescent risk behaviors and underpins the assessment and intervention of health risks in this demographic. The details of the study are described in detail in the previous study (48). Conducted triennially, this study utilizes data from the most recent survey, collected between April and June 2022. The survey involved a multi-stage random cluster sampling of 3,421 adolescents from grades 7 to 12 across four districts and counties in Hangzhou, Zhejiang Province. Participants completed a self-administered questionnaire covering various topics, including sociodemographic data, awareness of hypertension, physical activity, health and quality of life, smoking, alcohol use, mobile phone usage, dietary habits, weight control, hygiene practices, traffic and school safety, ear health, HIV/AIDS, and reproductive hygiene. Complete records were included.

#### 3.1.1 Sampling process

A multi-stage random cluster sampling was used in this study, and each stage was carried out according to the method of probability proportional sampling (PPS). In the first stage, four districts and counties of Hangzhou were randomly selected according to social and economic status; in the second stage, 11–13 middle school classes, 6–8 ordinary high school classes, and 6–8 non-ordinary high school classes were randomly selected from each district or county; in the third phase, students from all selected classes were invited to participate in the study.

#### 3.1.2 Sample size calculation

The sample size was calculated by using the following formula: $\;\text{deff}\times {\text{μ}}^{2}\times \text{P}\times \left(1-\text{P}\right)∕{\text{d}}^{2}$ . The prevalence rate of depression was 24.3% (49) and the rate of anxiety was 24.0% (50) in Chinese adolescent samples, and they were used as a measure of probability (P) (51), and the calculated maximum sample size is the final sample size. The design effect (deff) value was set at 3 and the relative error d was 0.03. The rate of invalid questionnaires was expected to be 20%, so the minimum sample size was calculated to be 2,825, and finally, 3,421 samples were collected. Due to the deletion of some outliers and questionnaires with incomplete important variables for the process of data analysis, our study included 3,307 participants in the final analysis.

### 3.2 Measurement

The study employed a self-administered questionnaire for data collection.

#### 3.2.1 Measurement of depression

The nine-item Patient Health Questionnaire (PHQ-9) was used to assess depressive symptoms (52). The response option on each item ranges from “not at all” (0 points) to “nearly every day” (3 points), yielding a total score from 0 to 27. The PHQ-9 has been translated into Chinese, and it has been validated and widely used across different Chinese samples (53–55). In this study, we further assessed the reliability and validity of this questionnaire.

#### 3.2.2 Measurement of anxiety

Anxiety symptoms were measured via the seven-item Generalized Anxiety Disorder Questionnaire (GAD-7) (56). The scale contains seven items, and each item is rated from 0 to 3, yielding a total score from 0 to 21. The Chinese version of GAD-7 has demonstrated reliability and validity among Chinese adolescents (57). In this study, we further assessed the reliability and validity of this questionnaire.

#### 3.2.3 Assessment of screen time

Different forms of screen time were assessed through the questions: “How long do you watch TV (including video games) each day on average on weekdays?”, “What is the amount of time you spend each day looking at the computer (not including for schoolwork), mobile phones, tablets, and game consoles?” respectively. Participants reported their average screen time per day on weekdays across seven categories: never, <1 h/day, 1 h/day, 2 h/day, 3 h/day, 4 h/day, and ≥5h/day. We set 2 hours as the cut-off value to judge whether teenagers’ screen time exceeds the standard (58), and adolescents who had an average of 2h or less of screen time per day on weekdays were coded as 0 whereas those who had more than 2h were coded as 1.

#### 3.2.4 Sleep duration

Sleep duration was gauged by asking students, “How many hours do you typically sleep on weekdays?” Participants were instructed to exclude awake time in bed from their reported sleep duration. Responses indicating sleep duration of less than 4 hours or more than 12 hours were considered outliers and excluded from the analysis.

#### 3.2.5 Covariates

Based on a comprehensive review of the existing literature and our understanding of the factors that may influence anxiety and depression symptoms in the study population, we selected the following covariates. The research collected demographic information including gender, age, height, and weight. Additionally, the study considered various potential risk factors that might influence anxiety and depression symptoms. These included academic performance/record, health status, number of close friends, days of physical activity per week, days of dread going to school, the number of times an item was stolen, and sleep duration. Age, height, weight, days of physical activity per week, and sleep duration were treated as continuous variables. Academic performance/record was divided into three categories, which were “Excellent”, “Middle”, and “Low”, and “Excellent” was taken as the reference. Health status was regarded as binary and was divided into “good” and “not good”, and “good” was taken as the reference. The number of close friends was divided into less than or equal to 2 and greater than 2, and less than or equal to 2 was taken as the reference. Dread going to school and items ever stolen or damaged were divided into ‘No’ and “Yes”, and “No” was taken as the reference. Anxiety and depression symptoms were classified as “No” and “Yes” based on whether they scored above 10 (10).

#### 3.2.6 Use of scales

In this study, the Physical Activity Scale, PHQ-9 scale, GAD-7 scale, Mobile Phone Addiction Index Scale (MPAI), and School Bullying Scale were used.

### 3.3 Statistical analysis

Mean and standard deviation were used to describe continuous variables, while categorical variables were presented as proportions. T-tests and Chi-square tests (χ2) were employed to assess statistical significance between groups with positive and negative outcomes for continuous and categorical variables, respectively. Two linear regression models were used to examine the relationship between screen time and the depression and anxiety symptom scores. Model 1 shows the results of the univariate regression analysis of these variables and the depression and anxiety symptom scores, while Model 2 explores the relationship between screen time and the scores of depression and anxiety symptoms after adjusting for age, gender, weight, height, academic performance, health status, number of close friends, days of physical activity per week, dread going to school, items ever stolen or damaged, and sleep duration. Additionally, we used the “mediation” package in RStudio and the “mediate” function to quantify the mediating role of sleep duration between screen time and depression and anxiety symptoms (59). All analyses were performed using R software version 4.2.1. In this study, the half coefficient and Cronbach’s α coefficient were used to evaluate the reliability of the scale, and we evaluated the construct validity of the questionnaires used in the study (60).

## 4 Results

### 4.1 Sample baseline characteristics

**Table 1** presents the sample’s baseline characteristics. The total sample size of our study was 3,421. We excluded participants with missing depression scores and anxiety symptoms scores and those with missing covariates. We finally included 3,307 participants, therefore, the response rate in this study was 96.67%. The average age of the teenagers in the study was 12–18 years, with an average age of 15.52 (± 1.62) years; 51.86% (1,715) of them were boys. Among these adolescents, 33.56% (1,109 individuals) spent more than the recommended 2 hours per day on electronic devices in the previous 12 months, and 17.46% (577 individuals) had more than 2 hours of television use per day. Within this group, the average depression symptom score was 5.942 ± 0.085, with a range of 0 to 27; and the average anxiety symptom score was 4.521 ± 0.076, with a range of 0 to 21.

**Table 1:** Baseline characteristics (N=3307).

Variable	N (%)
Overall	3,307
Age	15.524 ± 1.621
Gender	
Male	1,715 (51.860)
Female	1,592 (48.140)
Weight (kg)	57.659 ± 12.653
Height (cm)	167.049 ± 8.648
Academic performance/record	
Excellent	742 (22.437)
Middle	1,628 (49.229)
Low	937 (28.334)
Health status	
Good	3,039 (91.896)
Not good	268 (8.104)
Number of close friends	
≤ 2	811(24.524)
> 2	2,496(75.476)
Days of physical activity per week	4.336 ± 2.340
Dread going to school	
No	3,269 (98.851)
Yes	38 (1.149)
Items ever stolen or damaged	
No	3078 (93.075)
Yes	229 (6.925)
Depression score	5.942 ± 0.085
Anxiety score	4.521 ± 0.076
Electronic devices (h)	
≤2h	2196 (66.445)
>2h	1109 (33.555)
TV viewing time (h)	
≤2h	2728 (82.542)
>2h	577 (17.458)
Sleep duration (h)	7.982 ± 0.021

### 4.2 Reliability and validity test of scale

**Table 2** presents the reliability results of our scale. Cronbach’s α coefficient was 0.670 for the Physical Activity scale and 0.894 for the PHQ-9. Furthermore, the MPAI and School Bullying scales acquired 0.922 and 0.735 respectively. These scales all had coefficients above 0.6, and the coefficients of the split-half reliability test were also above 0.6. Thus, the reliability of the questionnaire was good.

**Table 2:** Reliability analysis.

Designation	Cronbach’s α coefficient	Split-half reliability
Physical Activity	0.670	0.670
PHQ-9	0.894	0.868
GAD-7	0.917	0.862
MPAI	0.922	0.855
School Bullying	0.735	0.739

In the Kaiser–Meyer–Olkin test, r=0.895, which was greater than 0.6, indicating that the data passed Bartlett’s test of sphericity and suggesting that the data possesses validity. Furthermore, Bartlett’s test of sphericity (χ2 = 165387.77, df=10440, P<0.001) indicated that the analysis model was appropriate. Therefore, factor analysis could be used to test the construct validity of the scale.

### 4.3 Overall association between screen time and mental disorders

**Table 3** displays the logistic regression results examining the association between screen time and a range of mental disorders, represented by *β* and 95% confidence intervals (CIs). After adjusting for all covariates, increased screen time was associated with increased scores for mental-related outcomes (electronic devices: *β*=0.601, 95%CI: 0.265 to 0.937 for depression symptoms and *β*=0.471, 95%CI: 0.159 to 0.784 for anxiety symptoms; television: *β*=0.751, 95%CI: 0.346 to 1.156 for depression symptoms and *β*=0.192, 95%CI: -0.185 to 0.569 for anxiety symptoms).

**Table 3:** Association between screen time and mental disorders.

	Depression symptom score				Anxiety symptom score			
	Model 1		Model 2		Model 1		Model 2	
	*β* (95% *CI*)	*P*	*β* (95% *CI*)	*P*	*β* (95% *CI*)	*P*	*β* (95% CI)	*P*
Electronic devices (h)								
≤2h	Ref		Ref		Ref		Ref	
>2h	1.241 (0.893,1.590)	<0.001	0.601 (0.265,0.937)	<0.001	0.747 (0.433,1.060)	<0.001	0.471 (0.159,0.784)	0.003
TV viewing time (h)								
≤2h	Ref		Ref		Ref		Ref	
>2h	0.987 (0.552,1.423)	<0.001	0.751 (0.346,1.156)	0.001	0.310 (-0.081,0.700)	0.120	0.192 (-0.185,0.569)	0.317
Sleep duration (h)	-1.017 (-1.148, -0.886)	<0.001	-0.832 (-0.953, -0.711)	<0.001	-0.885 (-1.002, -0.768)	<0.001	-0.757 (-0.869, -0.644)	<0.001
Age	0.223 (0.121,0.325)	<0.001	0.021 (-0.084,0.125)	0.698	0.098 (0.006,0.189)	0.036	-0.018 (-0.116,0.079)	0.711
Gender								
Male	Ref		Ref	<0.001	Ref		Ref	
Female	1.479 (1.151,1.807)	<0.001	1.260 (0.891,1.629)		1.381 (1.088,1.674)	<0.001	1.286 (0.942,1.629)	<0.001
Weight (kg)	-0.007 (-0.020,0.007)	0.331	-0.002 (-0.016,0.012)	0.852	-0.012 (-0.024, -0.001)	0.040	-0.002 (-0.015,0.011)	0.756
Height (cm)	-0.039 (-0.058, -0.020)	<0.001	0.002 (-0.021,0.026)	0.464	-0.040 (-0.057, -0.023)	<0.001	0.001 (-0.021,0.023)	0.947
Academic performance/record								
Excellent	Ref		Ref		Ref		Ref	
Middle	0.522 (0.105,0.938)	0.014	0.429 (0.061,0.797)	0.022	0.213 (-0.163,0.589)	0.267	0.210 (-0.132,0.553)	0.228
Low	2.090 (1.627,2.552)	<0.001	1.383 (0.971,1.795)	<0.001	1.159 (0.742,1.576)	<0.001	0.682 (0.299,1.066)	<0.001
Health status								
Good	Ref		Ref		Ref		Ref	
Not good	6.133 (5.563,6.704)	<0.001	4.742 (4.194,5.289)	<0.001	4.554 (4.033,5.074)	<0.001	3.471 (2.962,3.981)	<0.001
Number of close friends								
≤ 2	Ref		Ref		Ref		Ref	
> 2	-2.905 (-3.277, -2.532)	<0.001	-1.827 (-2.180, -1.474)	<0.001	-2.151 (-2.488, -1.814)	<0.001	-1.307 (-1.635, -0.979)	<0.001
Days of physical activity per week	-0.281 (-0.351, -0.211)	<0.001	-0.102 (-0.169, -0.035)	0.003	-0.147 (-0.210, -0.084)	<0.001	-0.005 (-0.067,0.058)	0.883
Dread going to school								
No	Ref		Ref		Ref		Ref	
Yes	4.318 (2.769,5.867)	<0.001	0.998 (-0.427,2.424)	0.170	3.892 (92.507,5.278)	<0.001	1.221 (-0.103,2.544)	0.071
Items ever stolen or damaged								
No	Ref		Ref		Ref		Ref	
Yes	2.962 (2.317,3.607)	<0.001	2.133 (1.524,2.742)	<0.001	2.772 (2.195,3.348)	<0.001	2.193 (1.627,2.758)	<0.001

Model 1: unadjusted.

Model 2: adjusted for age, gender, weight, height, academic performance, health status, number of close friends, days of physical activity per week, dread going to school, items ever stolen or damaged, and sleep duration.

### 4.4 Mediation effect of sleep duration on mental disorders

#### 4.4.1 Relationship between screen time and sleep duration

When controlling for covariates, there was a significant association between screen time and sleep duration (*β*=0.132, 95% CI: 0.036 to 0.228 for electronic device use; *β*=0.309, 95% CI: 0.194 to 0.424 for television use), as detailed in **Table 4**.

**Table 4:** Association between screen time and sleep duration.

	Sleep duration		
	*β*	95% *CI*	*P*
Electronic devices (h)			
≤2h	Ref	Ref	
>2h	0.132	0.036, 0.228	0.007
TV viewing time (h)			
≤2h	Ref	Ref	
>2h	0.309	0.194, 0.424	<0.001

Adjusted for age, gender, weight, height, academic performance, health status, number of close friends, days of physical activity per week, dread going to school, and items ever stolen or damaged.

#### 4.4.2 Relationship between sleep duration and mental disorders

**Table 5** illustrates the impact of sleep duration on mental disorders. The β for sleep duration in relation to depression symptoms was -0.832 (95% CI: -0.953 to -0.711, P<0.001) and for anxiety symptoms, it was -0.757 (95% CI: -0.869 to -0.644, P<0.001).

**Table 5:** Association between sleep duration and depression and anxiety symptom scores.

	Depression symptom score			Anxiety symptom score		
	*β*	95% *CI*	*P*	*β*	95% *CI*	*P*
Sleep duration	-0.832	-0.953, -0.711	<0.001	-0.757	-0.869, -0.644	<0.001

Adjusted for age, gender, weight, height, academic performance, health status, number of close friends, days of physical activity per week, dread going to school, items ever stolen or damaged, electronic devices, and TV viewing time.

#### 4.4.3 Mediation effect of sleep duration on the relationship between screen time and mental disorders

The relationship between screen time and mental disorders was partially mediated by sleep duration. For electronic device use, the mediation effect of sleep duration was -27.50% (P<0.001) for depression symptoms and -44.01% (P=0.018) for anxiety symptoms. For television use, the mediation effect of sleep duration was -42.70% (P=0.002), however, sleep duration did not have a statistically significant mediating effect on the relationship between screen time and anxiety symptoms (P=0.526), as shown in **Table 6**.

**Table 6:** The mediation effect of sleep on the relationship between screen time and depression and anxiety symptom scores.

	Direct effect *β* (95% *CI*)	Indirect effect *β* (95% *CI*)	Mediation effect	*P*
Electronic devices				
Depression symptoms	0.804 (0.467,1.139)	-0.173 (-0.257, -0.099)	-27.50%	<0.001
Anxiety symptoms	0.523 (0.220,0.822)	-0.162 (-0.240, -0.096)	-44.01%	0.018
TV viewing time				
Depression symptoms	0.981 (0.609,1.360)	-0.296 (-0.398, -0.198)	-42.70%	0.002
Anxiety symptoms	0.386 (0.042,0.744)	-0.274 (-0.372, -0.184)	>1	0.526

## 5 Discussion

This study revealed that 17.36% of the adolescents surveyed experienced depressive symptoms, and 11.67% experienced anxiety symptoms, with average scores of 5.94 ± 0.09 and 4.52 ± 0.08 respectively. The reliability and validity of the scales used in this study met the requirements, which means that our research has a certain scientific reliability. Notably, over one-quarter of the participants spent over 2h on electronic devices and television. The multivariate logistic regression results indicated a significant positive association between screen time and depression and anxiety symptom scores. Compared to people using electronic devices for 2h or less, people who used them more than 2h had higher depression and anxiety symptom scores (β=0.601,95%CI 0.265,0.937), which means that when the electronic device use increases from less than or equal to 2h to more than 2h, depression symptom scores increased by 0.601; and people who watched more than 2h of TV had higher depression symptom scores than those with less than 2h (β=0.751, 95%CI 0.346,1.156). These changes were both statistically significant. The results are consistent with previous studies in Canada, Norway, the United States, and Australia (61–63) that found that less screen time was strongly associated with fewer depressive symptoms and better health (64). The mechanism for the association between excessive screen time and depression symptoms may be direct or indirect. The direct approach can be seen in the following aspects: the impact of screen content, the disruption of interpersonal relationships, and the direct cognitive effect. These aspects can lead to reduced emotional stability and impulsive behavior (65). The indirect pathway can be observed through certain intermediate factors, such as sleep deprivation, dissatisfaction with weight, and being a victim of cyberbullying (66–68). The effect of electronic devices on anxiety symptoms scores was also positive, as those who used electronic devices for more than 2 hours had increased anxiety symptom scores by 0.471 (95%CI: 0.159,0.784) compared to those who watched less than 2 hours of video. Furthermore, we found that watching TV for more than 2h adds 0.192 (95%CI: -0.185,0.569) to anxiety symptom scores. In line with the social comparison theory and objectification theory (69), being exposed to unrealistic images that objectify the human body might lead to feelings of depression and anxiety symptoms (70).

Comparative research found that the depression symptom score was 7.80 ± 6.97 and the anxiety symptom score was 10.09 ± 5.16 in Canada (71), which were slightly higher than those of this study. Furthermore, a study showed that the median PHQ-9 Score was 2.5, 4, 6, and 7 for primary school, junior high school, technical secondary or high school, and junior college or undergraduate, respectively (72). The proportion of adolescents who spent time on screen-related activity exceeding 2h in our study was 33.56% for electronic devices and 17.46% for television, which was similar to that reported in a previous study in China (73). In recent years, many agencies have issued recommended screen time guidelines for children and teenagers. The American Academy of Pediatrics, the Australian Department of Health, the Canadian Pediatric Society, and the Chinese Physical Activity Guidelines for Children and Adolescents released in 2017 all recommend that children and adolescents should limit their daily screen time to less than 2 hours (74, 75). However, the proportion of adolescents who had excessive screen time in this study suggested that young people’s screen use is increasingly severe. A time-use diary study found that more time spent on social media screen time was related to a higher risk of self-harm (OR=1.13, 95% *CI* 0.22 to 0.50) and lower levels of self-esteem (*β*=-0.12, 95% *CI* -0.20 to -0.04) (29). Furthermore, weekend computer use was associated with a higher risk of post-traumatic stress disorder (PTSD) [odds ratio (OR) = 1.81] (76). The research results above point to more severe problems, and it is time to intervene with regard to teenagers’ screen time.

The multivariate logistic regression results from this study showed that girls had high depression and anxiety symptom scores. This finding is consistent with a large body of previous research. Biologically, hormonal fluctuations during puberty, which are more pronounced in girls, may contribute to increased emotional vulnerability (77). Interventions targeting girls could focus on providing education about hormonal changes, teaching healthy coping mechanisms for dealing with social pressures, and promoting positive body image. Those who had lower performance at school and had poorer health status were also at higher risk of depressive and anxiety symptoms. The link between lower school performance and higher risk of depressive and anxiety symptoms is multi-faceted. Poor academic performance can lead to feelings of inadequacy, low self-esteem, and stress. Students may worry about their future, parental expectations, and social standing among their peers. At the same time, pre-existing mental health issues can also interfere with concentration, memory, and motivation, thus affecting academic performance. A vicious cycle can be established, where poor mental health leads to poor grades, and poor grades further worsen mental health (78). Adolescents who had more friends had lower scores. Friends can provide emotional support, a sense of belonging, and opportunities for positive social interaction. They can also serve as a buffer against stress, helping adolescents to cope with difficult situations. However, it is important to note that the quality of friendships may be as important as the quantity. Having a few close, supportive friends may be more beneficial than having a large number of superficial acquaintances (79). This study found that physical activity can reduce depression symptoms levels. Exercise releases endorphins, which are natural mood-elevating chemicals in the body. It can also improve sleep quality, reduce stress, and increase self-esteem. In the context of adolescents, regular physical activity can provide a structured outlet for energy and emotions, as well as opportunities for social interaction (80). These findings align with prior findings (81), highlighting that these factors can be considered for intervention in adolescent mental disorders.

Our findings indicated that there was a negative association between sleep duration and mental health scores, which suggested that sleep was essential for the maintenance and protection of mental state, and a lack of sleep may lead to a host of mental problems. Similarly, sleep disorders such as insomnia, apnea, and circadian rhythm disturbances can lead to serious rates of chronic diseases (82). Moreover, sleep disorders have affected adolescents’ mental health and wellbeing (83). Improving sleep had a significant positive effect on overall mental health, depression, and anxiety, and in addition, improved sleep quality slightly reduced psychotic symptoms (84). This suggests that we should pay attention to the sleep problems of teenagers. During this stage, good sleep is not only crucial for the normal growth and development of the brain and body but also has a profound impact on cognitive function, emotional regulation, and social behavior. Unaddressed sleep problems can hinder teenagers’ academic performance, disrupt their social relationships, and even lay the foundation for long-term mental and physical health issues. By focusing on their sleep problems, we can help teenagers establish healthy sleep habits, which in turn will contribute to their overall wellbeing and set a positive trajectory for their future lives.

Another finding of this study was that increased screen time resulted in a slight increase in sleep time. Unlike previous research, which found a negative correlation between screen time and sleep time (85), this study found a positive correlation between the amount of time spent on electronic devices and television and sleep duration. This finding aligned with a report that TV use was associated with a later wake-up time (86), and this can also be explained by research that found that watching TV before going to bed was significantly correlated with sleep-wake transition disorder and excessive sleepiness disorder in children aged 5–6 years (87). These results suggest that increased screen time may trigger some compensatory sleep, but that this increased sleep is not necessarily healthy, which may lead to prolonged sleep onset latency, increased sleep time resistance, increased sleep anxiety, and increased overall sleep disorder levels (86). Furthermore, this may cause increased daytime sleepiness or fatigue in adolescents aged 12–17 (88, 89).

In this study, the influence of sleep was further studied as a mediator, and we uniquely calculated the mediating effect of sleep duration on the relationship between screen time and mental disorders, a connection not fully explored in previous research (73). Despite adjusting for sleep duration, a significant link between screen time and mental disorders persisted. However, the mediating effect was negative, which is different from previous studies that calculated a positive mediating effect (90). This may be due to excessive computer screen use leading to more compensatory sleep, and longer sleep duration itself can lower depression and anxiety symptom scores. However, such sleep may be compensatory and therefore not necessarily healthy. Overall, longer screen time was associated with poor mental disorder scores, and it partially reduced the mental disorder scores through compensatory sleep. Therefore, it is important to manage adolescents’ screen time and educate them on how to get healthy sleep, which may provide evidence for reducing the incidence of mental illness in adolescents. These findings have important implications for the prevention and treatment of adolescent mental disorders. School-based interventions can target at-risk groups such as girls, students with poor academic performance, and students with poor health. These interventions may include mental health education programs, social skills training, counseling services, and screen-equipment health education. Community-based initiatives, such as promoting physical activity and creating supportive social environments, can also play a key role. However, implementing these interventions requires collaboration between schools, families, healthcare providers, and policymakers. More research is needed to assess the effectiveness of these interventions and to develop more targeted and personalized approaches to improving adolescent mental health.

However, this study had some limitations. Because this study was cross-sectional, causation between screen time and mental disorders could not be established. Caution should be taken when generalizing our results to other populations, especially those with different cultural backgrounds and educational environments. While multi-stage sampling ensures the representativeness of the sample, the measurement of screen time relies on self-reporting, which may result in some inaccuracies. There are subjective biases in self-reported depression and anxiety symptoms scores, and individuals may have biased perceptions of their own emotional states, as some people may pay excessive attention to their negative emotions, while others may underestimate their emotional problems due to self-defense mechanisms. However, we conducted training before the students filled in the questionnaire, requiring the students to fill in the questionnaire objectively. In addition, students may mistake bedtime for sleep time, which was explained before students filled out the questionnaire. Finally, some covariates used binary variables. While this is common in similar studies, this can lead to some loss of information. In the future, more comprehensive cohort studies are needed to deepen the understanding of the relationship between screen time and mental disorders.

## 6 Conclusion

More screen time was associated with increased depression and anxiety symptom scores, and sleep may play a mediating role in this relationship.

## Data Availability

The original contributions presented in the study are included in the article/**Supplementary Material**. Further inquiries can be directed to the corresponding author.
