# Supplementary material for: Association between screen time and depressive and anxiety symptoms among Chinese adolescents
Source: Front Psychiatry. 2025 May 6;16:1428885. doi: 10.3389/fpsyt.2025.1428885 (PMC12089052; doi:10.3389/fpsyt.2025.1428885)
Supplement: Supplementary file 1 [file Table1.docx]

**Questionnaire on health risk factors of adolescents in Zhejiang Province**

**Part A: Demographic Information**

A1. What is your date of birth? (Gregorian calendar) (For example, if you were born in January 2002, enter 2002.01).

A2. What is your gender?

①Male

②Female

A3. What's your ethnicity?

①Han ethnicity

②Hui ethnicity

③She ethnicity

④Others

A4. What is the marital status of your parents?

① Married

② Divorced

③ Widowed

④ Separated

A5. What is your father's educational attainment?

① Primary school and below

② Junior high school

③ High school, technical secondary school or vocational school

④ Junior college or undergraduate

⑤ Master's degree and above

A6. What is your mother's educational attainment?

① Primary school and below

② Junior high school

③ High school, technical secondary school or vocational school

④ Junior college or undergraduate

⑤ Master's degree and above

A7. Are you an only child?

① Yes

② No

A8. What is your current accommodation situation?

① Living in the school dormitory

② Living at home with family (parents/grandparents)

③ Living at the home of other relatives

④ Renting a house outside the school

⑤ Others (specify) ___________

A9. What is the type of your household registration?

① Non-agricultural household registration (no rural homestead in the family)

② Agricultural household registration (with a rural homestead in the family)

A9a. Where is your household registration location?

① Local area

② Other places within the province

③ Other provinces

④ Unclear

A10. What is your current weight in kilograms? (kg) (Please note the difference: 1 kg = 2 Jin. For example, if your weight is 40.5 kg, fill in as 40.5).

A11. What is your current height in centimeters? (cm) (For example, if your height is 142 cm, fill in as 142.0).

A12. Compared with other students' families, what do you think your family's economic situation belongs to?

① Extremely poor

② Poor

③ Average

④ Affluent

⑤ Extremely affluent

**Part B: Awareness of Hypertension**

B1. Have you ever heard of hypertension?

① Yes

② No

B2. How long has it been since you had your blood pressure measured by a doctor or nurse last time?

① Never measured

② < 6 months

③ 6 months - 1-year

④ 1 - 2 years

⑤ 2 - 5 years

⑥ More than 5 years

B3. Have you ever been told by a doctor or nurse that you have hypertension?

① Yes

② No

③ Can't remember

**Part C: Physical Activity**

C1. In the past 7 days, on how many days did you engage in at least 60 minutes of activity each day (activities refer to any activity that makes your heart beat faster and causes shortness of breath, such as cycling, playing football, playing basketball, kicking shuttlecocks, etc.)?

① None

② 1-day

③ 2 days

④ 3 days

⑤ 4 days

⑥ 5 days

⑦ 6 days

⑧ 7 days

C1a. In the past 7 days, on how many days did you walk or ride a bicycle (excluding electric vehicles) to or from school?

① None

② 1-day

③ 2 days

④ 3 days

⑤ 4 days

⑥ 5 days

⑦ 6 days

⑧ 7 days

C2. In the past 7 days, on how many days did you do muscle - strengthening activities (such as push - ups, sit - ups, lifting dumbbells, weightlifting, etc.)?

① None

② 1-day

③ 2 days

④ 3 days

⑤ 4 days

⑥ 5 days

⑦ 6 days

⑧ 7 days

C3. Generally, on how many days of a week do you have physical education classes?

① None

② 1-day

③ 2 days

④ 3 days

⑤ 4 days

⑥ 5 days

⑦ 6 days

⑧ 7 days

C3a. In the past 7 days, on how many days did you do household chores (such as cleaning, tidying up, raising animals)?

① None

② 1-day

③ 2 days

④ 3 days

⑤ 4 days

⑥ 5 days

⑦ 6 days

⑧ 7 days

C4. From Monday to Friday, how long do you watch TV on average each day (including TV games like X - BOX)?

① Don't watch TV

② < 1 hour/day

③ 1 - 2 hours/day

④ 2 - 3 hours/day

⑤ 3 - 4 hours/day

⑥ 4 - 5 hours/day

⑦ ≥ 5 hours/day

C5. From Monday to Friday, how long do you usually spend on playing with computers, mobile phones, tablets, game consoles on average each day (excluding using the computer for school assignments)?

① Don't play or use the computer only for school assignments

② < 1 hour/day

③ 1 - 2 hours/day

④ 2 - 3 hours/day

⑤ 3 - 4 hours/day

⑥ 4 - 5 hours/day

⑦ ≥ 5 hours/day

**Part D: Health Status and Quality of Life**

D1. Which of the following best describes your self-perceived health status?

① Excellent

② Good

③ Average

④ Poor

⑤ Very poor

⑥ Unsure

D2. In the past 12 months, have you ever felt lonely?

① Never

② Occasionally

③ Sometimes

④ Often

⑤ Always

D3. In the past 12 months, have you ever had insomnia due to worrying about something?

① Never had insomnia

② Occasionally had insomnia

③ Sometimes had insomnia

④ Often had insomnia

⑤ Always had insomnia

D4. In the past 12 months, have you ever felt so sad or desperate for two consecutive weeks or more that you stopped your daily activities?

① Yes

② No

D5. In the past 12 months, have you ever seriously considered suicide?

① Yes

② No

D6. In the past 12 months, have you ever made a suicide plan?

① Yes

② No

D7. In the past 12 months, have you ever attempted suicide?

① No

② Once

③ ≥2 times

D8. In the past 12 months, if you have ever attempted suicide, did you get injured and must go to the hospital for treatment?

① I have not attempted suicide in the past 12 months

② I attempted suicide and went to the hospital for treatment

③ I attempted suicide but did not go to the hospital

D9. What do you think of your academic performance in the class?

① Excellent

② Average

③ Below average

D10. In the past 30 days, how many hours did you sleep on average per day? (including naps)

D10a. In the past 30 days, how much later did you wake up on weekends compared to weekdays?

① Wake - up time remained the same

② 30 minutes - 1 hour later per day

③ 1 - 2 hours later per day

④ 2 - 3 hours later per day

⑤ 3 - 4 hours later per day

⑥ 4 - 5 hours later per day

⑦ ≥ 5 hours later per day

D10b. In the past 30 days, how much later did you go to bed on weekends compared to weekdays?

① Bed - time remained the same

② 30 minutes - 1 hour later

③ 1 - 2 hours later

④ 2 - 3 hours later

⑤ 3 - 4 hours later

⑥ 4 - 5 hours later

⑦ ≥ 5 hours later

D11. In the past 30 days, how many days did you skip classes? (Skipping classes means subjectively asking for leave for various reasons or not going to school without a reason because you don't want to attend class)

① None

② 1 - 2 days

③ 3 - 5 days

④ 6 - 9 days

⑤ 10 days or more

D12. In the past 12 months, how many times have you run away from home?

① None

② 1-time

③ 2 times

④ 3 times

⑤ 4 times

⑥ 5 times

⑦ 6 times or more

D13. How many close friends do you have?

① None

② 1

③ 2

④ 3 or more

D14. The following are descriptions of possible feelings you may have had in the past two weeks. Please choose the option that best suits your actual situation according to the frequency of these feelings.

a. Lack of energy or interest in doing things

① None

② For a few days

③ More than half of the days

④ Almost every day

b. Feel down, depressed or hopeless

① None

② For a few days

③ More than half of the days

④ Almost every day

c. Have trouble falling asleep, staying asleep or sleep too much

① None

② For a few days

③ More than half of the days

④ Almost every day

d. Feel tired or lack energy

① None

② For a few days

③ More than half of the days

④ Almost every day

e. Lose appetite or overeat

① None

② For a few days

③ More than half of the days

④ Almost every day

f. Think you're bad or a failure, or have let your family down

① None

② For a few days

③ More than half of the days

④ Almost every day

g. Have difficulty concentrating on things

① None

② For a few days

③ More than half of the days

④ Almost every day

h. Move or speak so slowly that others notice, or conversely - be fidgety or restless and move around more than usual

① None

② For a few days

③ More than half of the days

④ Almost every day

j. Have thoughts of wanting to die or hurting yourself in some way

① None

② For a few days

③ More than half of the days

④ Almost every day

D15. In the past two weeks, how often did the following symptoms occur in your life?
a. Feeling nervous, anxious or on edge

① None

② For a few days

③ More than half of the days

④ Almost every day

b. Not being able to stop or control worrying

① None

② For a few days

③ More than half of the days

④ Almost every day

c. Worrying a lot about a variety of things

① None

② For a few days

③ More than half of the days

④ Almost every day

d. Having difficulty relaxing

① None

② For a few days

③ More than half of the days

④ Almost every day

e. Being unable to sit still because of restlessness

① None

② For a few days

③ More than half of the days

④ Almost every day

f. Becoming easily annoyed or irritable

① None at all

② For a few days

③ More than half of the days

④ Almost every day

g. Feeling afraid as if something awful might happen

① None

② For a few days

③ More than half of the days

④ Almost every day

**The following are questions regarding serious injuries. A serious injury refers to any situation where, due to various causes such as being cut by a knife, traffic accidents, burns, falls, being bitten or scratched by an animal, drowning, poisoning, getting injured during sports, or getting into a fight with others, you need to receive medical treatment from a doctor or rest for at least one day. If an injury occurred in the past 12 months but you did not receive medical treatment or the rest time was less than one day, it will not be counted as a serious injury here.**

D16. In the past 12 months, how many times have you suffered serious injuries?

① None

② 1-time

③ 2 times

④ 3 times

⑤ 4 times

⑥ 5 times

⑦ ≥6 times

**D17 - D19 questions are to understand the HPV vaccine (also known as human papillomavirus vaccine) vaccination situation among girls. Vaccination with the HPV vaccine can prevent cervical cancer. (For girls only)**

D17. Have you ever heard of the HPV vaccine?

① Yes

② No

D18. Have you completed the full course of HPV vaccination?

① Not vaccinated

② Completed the full course of vaccination

③ Vaccinated, but not completed the full course

D19. What type of HPV vaccine did you receive?

① Not vaccinated

② Bivalent

③ Quadrivalent

④ Nonavalent

⑤ Vaccinated, but don't know the type

**Part E: Smoking**

E1. Do you think smoking has an impact on health?

① Harmless

② Slightly harmful

③ Moderately harmful

④ Seriously harmful

⑤ Don't know

E2. Do you think passive smoking (i.e., inhaling the smoke exhaled by other smokers) has an impact on health?

① Harmless

② Slightly harmful

③ Moderately harmful

④ Seriously harmful

⑤ Unclear E3. Among the people you live with, are there any smokers?

① No

② There are male smokers

③ There are female smokers

④ There are both male and female smokers

E4. In the past 7 days, on how many days did others smoke in front of you?

① None

② 1-day

③ 2 days

④ 3 days

⑤ 4 days

⑥ 5 days

⑦ 6 days

⑧ 7 days

E6. Have you ever smoked a cigarette, even just one or two puffs?

① Yes

② No

E6a. In total, how many cigarettes have you smoked from birth until now?

① I've never smoked a cigarette, not even one or two puffs

② One or two puffs, but less than 1 cigarette

③ 1 cigarette

④ 2 - 5 cigarettes

⑤ 6 - 15 cigarettes (half a pack)

⑥ 16 - 25 cigarettes (1 pack)

⑦ 26 - 99 cigarettes (1 - 5 packs)

⑧ ≥100 cigarettes (more than 5 packs)

E7. How old were you (please answer in full years) when you first tried smoking a cigarette?

① Never smoked

② ≤8 years old

③ 9 or 10 years old

④ 11 or 12 years old

⑤ 13 or 14 years old

⑥ 15 or 16 years old

⑦ ≥17 years old
E8. What was the main motivation for you to smoke? (Choose only one option)

① Never smoked

② Curiosity, wanted to give it a try

③ Influence of classmates

④ To relieve stress

⑤ Encouraged by adults

⑥ Thought smoking looked cool and imitated the smoking gesture

⑦ For setting off fireworks

⑧ Others _________

E9. How old were you (please answer in full years) when you first smoked a whole cigarette?

① Never smoked

② ≤8 years old

③ 9 or 10 years old

④ 11 or 12 years old

⑤ 13 or 14 years old

⑥ 15 or 16 years old

⑦ ≥17 years old

E10. In the past 30 days, on how many days did you smoke cigarettes?

① I didn't smoke cigarettes in the past 30 days

② 1 - 2 days

③ 3 - 5 days

④ 6 - 9 days

⑤ 10 - 19 days

⑥ 20 - 29 days

⑦ 30 days

E11. In the past 30 days, how did you usually get the cigarettes you smoked?

① I didn't smoke cigarettes in the past 30 days

② Bought from a store

③ Bought from a peddler

④ Bought from a vending machine

⑤ Asked someone to buy for me

⑥ Asked others for or were given by others

⑦ Stole them

⑧ Others

E12. On the days you smoked in the past 30 days, how many cigarettes did you smoke on average per day?

① Didn't smoke in the past 30 days

② Less than 1 cigarette

③ 1 cigarette per day

④ 2 - 5 cigarettes per day

⑤ 6 - 10 cigarettes per day

⑥ 11 - 20 cigarettes per day

⑦ More than 20 cigarettes per day

E13. Have you ever tried other tobacco products besides cigarettes (such as water pipes / cigars / pipes, etc.)?

① No

② Yes

E13a. In the past 30 days, have you used other tobacco products besides cigarettes (such as water pipes / cigars / pipes, etc.)?

① No

② Yes

E14. In the past 12 months, have you tried to quit smoking?

① Never smoked or only smoked one or two puffs

② Yes, tried to quit smoking

③ Didn't try to quit smoking

**The following questions are about e - cigarettes. E - cigarettes, similar in appearance to cigarettes, are electronic devices driven by batteries to simulate the smoking process. The vapor produced by e - cigarettes usually contains nicotine.**

E15. Have you heard of e - cigarettes before?

① Yes

② No

E16. In the past 30 days, on how many days did you use e - cigarettes?

① 0 days

② 1 - 2 days

③ 3 - 5 days

④ 6 - 9 days

⑤ 10 - 19 days

⑥ 20 - 29 days

⑦ 30 days

E17. From birth until now, how many e - cigarette cartridges have you used in total?

① 0 ② Less than 1

③ 1 - 2

④ 3 - 10

⑤ 11 - 20

⑥ 21 - 50

⑦ More than 50

**Part F: Alcohol Consumption**

**Alcohol consumption here refers to drinking at least one glass of alcohol. One glass of alcohol is equivalent to half a bottle / one can of beer, a small cup of Chinese liquor, a glass of wine or yellow rice wine. Just taking a sip or tasting the flavor of alcohol does not count as drinking.**

F1. In total, from birth until now, how many days have you consumed alcohol?

① Never drank or just had a taste

② 1 or 2 days

③ 3 - 9 days

④ 10 - 19 days

⑤ 20 - 39 days

⑥ 40 - 99 days

⑦ 100 days or more

F2. How old were you (please answer in full years) when you first drank alcohol?

① Never drank or just had a taste

② ≤8 years old

③ 9 or 10 years old

④ 11 or 12 years old

⑤ 13 or 14 years old

⑥ 15 or 16 years old

⑦ ≥17 years old

F3. In the past 30 days, on how many days did you consume alcohol?

① Never drank or just had a taste

② 1 - 2 days

③ 3 - 5 days

④ 6 - 9 days

⑤ 10 - 19 days

⑥ 20 - 29 days

⑦ 30 days

F4. (This question is for boys only) In the past 30 days, on how many days did you drink at least 5 glasses of alcohol within 1 - 2 hours?

① Never drank or didn't drink 5 glasses

② 1-day

③ 2 days

④ 3 - 5 days

⑤ 6 - 9 days

⑥ 10 - 19 days

⑦ ≥20 days

F5. (This question is for girls only) In the past 30 days, on how many days did you drink at least 4 glasses of alcohol within 1 - 2 hours?

① Never drank or didn't drink 4 glasses

② 1-day

③ 2 days

④ 3 - 5 days

⑤ 6 - 9 days

⑥ 10 - 19 days

⑦ ≥20 days

F6. In the past 30 days, how did you usually obtain the alcohol you drank? (Choose only one option)

① Never drank alcohol

② Bought it with my own money

③ Took it from home

④ Someone treated me

⑤ Obtained it through other means _________

F7. How many times have you been drunk (being drunk is manifested as unsteady gait, drowsiness, vomiting or slurred speech)?

① Never been drunk

② 1 - 2 times

③ 3 - 9 times

④ 10 times or more

F8. How old were you (please answer in full years) when you first got drunk (being drunk is manifested as unsteady gait, drowsiness, vomiting or slurred speech)?

① Never been drunk

② ≤7 years old

③ 8 - 9 years old

④ 10 - 11 years old

⑤ 12 - 13 years old

⑥ 14 - 15 years old

⑦ ≥16 years old

**Part G: Mobile Phone Use**

G1. Do you have your own mobile phone?

① Yes

② No

G2. Do you have the habit of putting your mobile phone beside your pillow when you sleep?

① I don't have a mobile phone.

② I have a mobile phone, but I usually don't put it beside my pillow.

③ I have a mobile phone and usually put it beside my pillow.

G3. From Monday to Friday, how long do you usually spend on playing with your mobile phone on average each day (excluding using the mobile phone for school assignments)?

① Don't play with the mobile phone or use it only for school assignments

② < 1 hour/day

③ 1 - 2 hours/day

④ 2 - 3 hours/day

⑤ 3 - 4 hours/day

⑥ 4 - 5 hours/day

⑦ ≥ 5 hours/day

G4. On Saturdays and Sundays, how long do you usually spend on playing with your mobile phone on average each day (excluding using the mobile phone for school assignments)?

① Don't play with the mobile phone or use it only for school assignments

② < 1 hour/day

③ 1 - 2 hours/day

④ 2 - 3 hours/day

⑤ 3 - 4 hours/day

⑥ 4 - 5 hours/day

⑦ ≥ 5 hours/day

G5. In the past 12 months, have you had the following feelings or experiences?

a. Have you ever been told that you spend too much time using your mobile phone?

① Never

② Occasionally

③ Sometimes

④ Often

⑤ Always

b. Have your friends/family members complained that you always use your mobile phone?

① Never

② Occasionally

③ Sometimes

④ Often

⑤ Always

c. Have you ever tried to hide from others how much time you spend using your mobile phone?

① Never

② Occasionally

③ Sometimes

④ Often

⑤ Always

d. Have you found that you spend more time using your mobile phone than you expected?

① Never

② Occasionally

③ Sometimes

④ Often

⑤ Always

e. Do you always feel that you don't have enough time to use your mobile phone?

① Never

② Occasionally

③ Sometimes

④ Often

⑤ Always

f. Have you tried to spend less time on your mobile phone but failed?

① Never

② Occasionally

③ Sometimes

④ Often

⑤ Always

g. Have you sacrificed your sleep time because of using your mobile phone?

① Never

② Occasionally

③ Sometimes

④ Often

⑤ Always

h. Do you start to worry that you'll miss calls or online messages from friends if you don't check your phone for a certain period?

① Never

② Occasionally

③ Sometimes

④ Often

⑤ Always

i. Do you become anxious if you don't check text messages, go online, or turn on your phone for a while?

① Never

② Occasionally

③ Sometimes

④ Often

⑤ Always

j. Do you find it difficult to turn off your phone?

① Never

② Occasionally

③ Sometimes

④ Often

⑤ Always

k. Do you feel lost without your mobile phone?

① Never

② Occasionally

③ Sometimes

④ Often

⑤ Always

m. When you feel isolated, do you use your mobile phone to communicate with others?

① Never

② Occasionally

③ Sometimes

④ Often

⑤ Always

n. When you feel lonely, have you used your mobile phone to communicate with others?

① Never

② Occasionally

③ Sometimes

④ Often

⑤ Always

p. When you feel down, have you used your mobile phone to relieve your feelings of disappointment?

① Never

② Occasionally

③ Sometimes

④ Often

⑤ Always

q. Have you been delayed in other matters because you were busy using your mobile phone, and thus caused trouble?

① Never

② Occasionally

③ Sometimes

④ Often

⑤ Always

r. Is the time you spend on your mobile phone the direct cause of a decline in your initiative?

① Never

② Occasionally

③ Sometimes

④ Often

⑤ Always

s. Do you sometimes prefer to use your mobile phone rather than deal with other more urgent problems?

① Never

② Occasionally

③ Sometimes

④ Often

⑤ Always

G6. After school, how long do you spend on average each day to complete your written homework?

① No homework

② ≤30 minutes/day

③ 31 - 59 minutes/day

④ ≥1 hour/day

⑤ ≥1.5 hours/day

⑥ ≥2 hours/day

G7. In the last semester (before the "Double Reduction" policy), how long did you spend on average each day reading?

① No reading

② ≤30 minutes/day

③ 31 - 59 minutes/day

④ ≥1 hour/day

⑤ ≥2 hours/day

⑥ ≥3 hours/day

G8. In this semester (after the "Double Reduction" policy), how long do you spend on average each day reading?

① No reading

② ≤30 minutes/day

③ 31 - 59 minutes/day

④ ≥1 hour/day

⑤ ≥2 hours/day

⑥ ≥3 hours/day

**Part J: Eating Habits**

J1. In the past 7 days, on how many days did you have breakfast?

① None

② 1-day

③ 2 days

④ 3 days

⑤ 4 days

⑥ 5 days

⑦ 6 days

⑧ 7 days

J2. In the past 30 days, how many times did you usually eat fruits per day?

① None

② Less than 1 time/day

③ 1 time/day

④ 2 times/day

⑤ 3 times/day

⑥ 4 times/day

⑦ ≥5 times/day

J3. In the past 30 days, how many times did you usually eat vegetables per day?

① None

② Less than 1 time/day

③ 1 time/day

④ 2 times/day

⑤ 3 times/day

⑥ 4 times/day

⑦ ≥5 times/day

J4. In the past 30 days, on how many days of each week did you usually drink milk?

① Never drank it

② Less than 1-day

③ 1 - 2 days

④ 3 - 4 days

⑤ 5 - 7 days

J5. In the past 7 days, how many times did you usually drink carbonated beverages (such as Coke, Sprite, Fanta or Marinda, etc.)?

① Didn't drink any

② 1 - 3 times a week

③ 4 - 6 times a week

④ Once a day

⑤ Twice a day

⑥ Three times a day

⑦ Four times a day or more

J5a. In the past 7 days, how many times did you usually eat puffed snacks (such as potato chips, prawn crackers or snow cakes, etc.)?

① Didn't eat any

② 1 - 3 times a week

③ 4 - 6 times a week

④ Once a day

⑤ Twice a day

⑥ Three times a day

⑦ Four times a day or more

J6. In the past 7 days, on how many days did you have Western - style fast food, such as McDonald's, KFC, etc. (fast - food restaurants with hamburgers, fried chicken and fries)?

① None

② 1-day

③ 2 days

④ 3 days

⑤ 4 days

⑥ 5 days

⑦ 6 days

⑧ 7 days

**Part K: Weight Control**

K1. Which of the following do you think your weight belongs to?

① Very light

② A bit light

③ Moderate

④ A bit heavy

⑤ Very heavy

K2. Are you currently controlling your weight?

① Yes

② No

K3. In the past 30 days, have you taken laxatives to control or reduce your weight?

① Yes

② No

K3a. In the past 30 days, have you induced vomiting to reduce your weight?

① Yes

② No

K4. In the past 30 days, without a doctor's guidance, have you taken diet pills to control or reduce your weight?

① Yes

② No

K5. In the past 30 days, have you controlled or reduced your weight by dieting or eating low - calorie foods?

① Yes

② No

K6. In the past 30 days, have you refrained from eating for at least 24 hours to control or reduce your weight?

① Yes

② No

K7. In the past 30 days, have you increased your physical activity to control or reduce your weight?

① Yes

② No

**Part L: Hygiene Habits**

L1. In the past 30 days, how many times did you usually brush your teeth each day?

① Didn't brush teeth

② < 1 time/day

③ 1 time/day

④ 2 times/day

⑤ 3 times/day

⑥ 4 times/day or more

L2. In the past 30 days, did you wash your hands before eating?

① Never washed hands

② Occasionally washed hands

③ Sometimes washed hands

④ Washed hands in most cases

⑤ Always washed hands

L3. In the past 30 days, did you wash your hands after using the toilet?

① Never washed hands

② Occasionally washed hands

③ Sometimes washed hands

④ Washed hands in most cases

⑤ Always washed hands

L4. In the past 30 days, did you use soap, toilet soap or hand sanitizer when washing your hands?

① Never used

② Occasionally used

③ Sometimes used

④ Used in most cases

⑤ Always used

L5. What is your current eyesight (eyesight refers to the naked - eye vision without wearing glasses)?

① Above 5.0

② 4.9 - 4.7

③ 4.6 - 4.4

④ Below 4.3

⑤ Don't know

L6. Currently, do you wear glasses (including contact lenses)?

① Don't wear

② Wear all the time

③ Only wear in class

④ Used to wear, but not now

L7. How often do you do eye exercises?

① 1 - 2 times a day

② 4 - 5 times a week

③ 2 - 3 times a week

④ ≤1 time a week

L8. Are your parents nearsighted?

① Neither is nearsighted

② One of them is nearsighted

③ Both are nearsighted

④ Not sure

L9. Do you have the habit of reading while lying down?

① Yes

② No

**Part M: Traffic Safety**

M1. In the past 12 months, have you ridden a bicycle?

① Never ridden a bicycle

② Ridden occasionally

③ Ridden sometimes

④ Ridden frequently

M2. In the past 12 months, when riding a bicycle, have you had experiences of running red lights, occupying motor vehicle lanes, carrying passengers, not using hand signals when turning, or riding in the opposite direction (traffic violations)?

① Never ridden a bicycle

② Did this occasionally

③ Did this sometimes

④ Did this frequently

⑤ Ridden a bicycle but without traffic violations

M3. In the past 30 days, when taking a car or taxi, did you fasten your seatbelt?

① Didn't sit in the front seat of a car or taxi

② Never fastened

③ Fastened occasionally

④ Fastened sometimes

⑤ Fastened frequently

M4. In the past 30 days, have you driven a motor vehicle? (Motor vehicles do not include pedal - powered bicycles, motor - assisted bicycles, electric bicycles, or motor - driven wheelchairs for the disabled)

① Yes

② No (If you choose "2", go to Part N)

M5. What type of motor vehicle did you drive?

① Motorcycle

② Tractor

③ Car

④ Truck

⑤ Jeep, minibus, station wagon (with less than 10 seats)

⑥ Bus (with more than 10 seats)

⑦ Others _______

M6. In the past 30 days, have you had the experience of driving after drinking?

① Yes

② No

③ Not sure

**Part N: Related Behaviors**

N1. In the past 12 months, how many times have you been in a fight?

① None

② 1-time

③ 2 - 3 times

④ 4 - 5 times

⑤ 6 - 7 times

⑥ 8 - 9 times

⑦ 10 - 11 times

⑧ 12 times or more

N2. In the past 12 months, how many times have you been injured in a fight and gone to the hospital for treatment?

① None

② 1-time

③ 2 - 3 times

④ 4 - 5 times

⑤ 6 - 7 times

⑥ 8 - 9 times

⑦ 10 - 11 times

⑧ 12 times or more

N3. In the past 30 days, on how many days did you carry weapons such as knives, daggers, or clubs for self - defense or fighting?

① None

② 1-day

③ 2 - 3 days

④ 4 - 5 days

⑤ 6 days or more

N4. In the past 30 days, on how many days did you not go to school because you felt unsafe on the way to and from school?

① None

② 1-day

③ 2 - 3 days

④ 4 - 5 days

⑤ 6 days or more

N5. In the past 12 months, how many times have you been threatened with weapons such as knives, daggers, or clubs or been injured on campus?

① None

② 1-time

③ 2 - 3 times

④ 4 - 5 times

⑤ 6 - 7 times

⑥ 8 - 9 times

⑦ 10 - 11 times

⑧ 12 times or more

N6. In the past 12 months, how many times have your clothes, textbooks, books, etc. been stolen or deliberately damaged on campus?

① None

② 1-time

③ 2 - 3 times

④ 4 - 5 times

⑤ 6 - 7 times

⑥ 8 - 9 times

⑦ 10 - 11 times

⑧ 12 times or more

N7. In the past 30 days, have you experienced the following forms of bullying on campus or around the campus?

**Bullying refers to being maliciously and impolitely teased and attacked verbally or behaviorally by others, making the victim feel annoyed or uncomfortable, and suffering mental or physical trauma; it does not include friendly jokes or actions.**

N7a. Being maliciously made fun of

① None

② Once

③ ≥ Twice

N7b. Having money or property demanded from you

① None

② Once

③ ≥ Twice

N7c. Being deliberately excluded from group activities or isolated

① None

② Once

③ ≥ Twice

N7d. Being threatened or intimidated

① None

② Once

③ ≥ Twice

N7e. Being hit, kicked, pushed, shoved, or locked in a room

① None

② Once

③ ≥ Twice

N7f. Being made fun of because of physical defects or appearance

① None

② Once

③ ≥ Twice

N7g. In the past 30 days, has anyone maliciously made fun of, insulted, threatened, intimidated you, or spread rumors, images, or videos about you through electronic media?

① None

② Once

③ ≥ Twice

N8. In the past 30 days, have you bullied classmates or other people?

① None

② Once

③ ≥ Twice

**Part W: Unhealthy Ear - using Behaviors**

The following are some questions about improper ear - using behaviors.

W1. Do you use headphones (for at least 30 minutes continuously)?

① No

② Yes

W2. In the past 7 days, how many minutes did you use headphones on average each day? minutes

W3. In the past 7 days, how many times did you use headphones continuously for more than 60 minutes?

① Never

② 1 - 2 times

③ 3 - 4 times

④ 5 - 6 times

⑤ Once a day

⑥ ≥ Twice a day

W4. In the past 7 days, did you use headphones for a long time (for at least 30 minutes continuously) in a noisy environment (such as a station, subway, mall, etc. where you need to turn up the volume)?

① Never

② 1 - 2 times

③ 3 - 4 times

④ 5 - 6 times

⑤ Once a day

⑥ ≥ Twice a day

W5. In the last month, have you felt that you can't hear as clearly as before?

① No

② Yes

**Part P: AIDS (Acquired Immune Deficiency Syndrome)**

**The following are questions about AIDS. Please answer them.**

P1a. Is AIDS a serious incurable infectious disease?

① Yes

② No

③ Don't know

P1b At present, the prevalence of AIDS among young students in China is showing a rapid growth trend. The main mode of transmission is male same-sex sexual behavior, followed by heterosexual sexual behavior. Is that right?

①Yes

②No

③Don't know

P1c. Can you tell if a person is infected with AIDS just by looking at them?

①Yes

②No

③Don't know

P1d. Can you get infected with AIDS through daily life and study contacts?

①Yes

②No

③Don't know

P1e. Can insisting on the correct use of condoms reduce the risk of AIDS infection and transmission?

①Yes

②No

③Don't know

P1f. Does the use of new drugs (such as methamphetamine, ecstasy, ketamine, etc.) increase the risk of AIDS infection?

①Yes

②No

③Don't know

P1g. Should people actively seek AIDS testing and counseling after engaging in high-risk behaviors (such as sharing needles for drug use / unsafe sex, etc.)?

①Yes

②No

③Don't know

P1h. Are the rights of AIDS virus carriers to marry, get employed and enter school protected by Chinese law?

①Yes

②No

③Don't know

P2. In the last school year, did the teacher give a course on how to prevent AIDS?

①Yes

②No

**S part: Reproductive health**

**Questions S1 and S2 are for girls only.**

S1. Have you ever had your period?

①Yes

②No

③Don't know

If yes, when was your first period?

S2. Do you feel that your breasts have started to develop?

①Yes

②No

③Don't know

**S3 and S4 are for boys only.**

S3. Do you feel that you have started to experience voice - change?

① Yes

② No

③ Don't know

S4. Have you ever had a nocturnal emission?

① Yes

② No

③ Don't know

S5. Have you ever had sexual intercourse? (Sexual intercourse refers to the contact of both parties' sexual organs.)

① Yes

② No

S6. At what age did you have your first sexual intercourse? (Please answer in full years of age.)

① Never had sexual intercourse

② ≤11 years old

③ 12 years old

④ 13 years old

⑤ 14 years old

⑥ 15 years old

⑦ 16 years old

⑧ 17 years old ⑨ ≥18 years old

S7. With how many people have you had sexual intercourse?

① Never had sexual intercourse

② 1-person

③ 2 people

④ 3 or more people

S8. Did you or your partner use a condom during the most recent sexual intercourse?

① Never had sexual intercourse

② Used a condom during the most recent sexual intercourse

③ Didn't use a condom during the most recent sexual intercourse

S9. Have you ever been pregnant or made your partner pregnant?

① Never had sexual intercourse

② Had sexual intercourse but never been pregnant or made the partner pregnant

③ Have been pregnant or made the partner pregnant

**The questionnaire is completed. Thank you for your cooperation!**
